# Innovative Strategy for MicroRNA Delivery in Human Mesenchymal Stem Cells via Magnetic Nanoparticles

**DOI:** 10.3390/ijms140610710

**Published:** 2013-05-23

**Authors:** Anna Schade, Evgenya Delyagina, Dorothee Scharfenberg, Anna Skorska, Cornelia Lux, Robert David, Gustav Steinhoff

**Affiliations:** Reference and Translation Center for Cardiac Stem Cell Therapy (RTC), Department of Cardiac Surgery, University of Rostock, Schillingallee 35, 18057 Rostock, Germany; E-Mails: anna.schade@med.uni-rostock.de (A.S.); evgenya.delyagina@med.uni-rostock.de (E.D.); sndoro@web.de (D.S.); anna.skorska@med.uni-rostock.de (A.S.); cornelia.lux@med.uni-rostock.de (C.L.); davidrob@med.uni-rostock.de (R.D.)

**Keywords:** magnetic nanoparticles, microRNA, polyethylenimine, mesenchymal stem cells, non-viral carrier

## Abstract

Bone marrow derived human mesenchymal stem cells (hMSCs) show promising potential in regeneration of defective tissue. Recently, gene silencing strategies using microRNAs (miR) emerged with the aim to expand the therapeutic potential of hMSCs. However, researchers are still searching for effective miR delivery methods for clinical applications. Therefore, we aimed to develop a technique to efficiently deliver miR into hMSCs with the help of a magnetic non-viral vector based on cationic polymer polyethylenimine (PEI) bound to iron oxide magnetic nanoparticles (MNP). We tested different magnetic complex compositions and determined uptake efficiency and cytotoxicity by flow cytometry. Additionally, we monitored the release, processing and functionality of delivered miR-335 with confocal laser scanning microscopy, real-time PCR and live cell imaging, respectively. On this basis, we established parameters for construction of magnetic non-viral vectors with optimized uptake efficiency (~75%) and moderate cytotoxicity in hMSCs. Furthermore, we observed a better transfection performance of magnetic complexes compared to PEI complexes 72 h after transfection. We conclude that MNP-mediated transfection provides a long term effect beneficial for successful genetic modification of stem cells. Hence, our findings may become of great importance for future *in vivo* applications.

## 1. Introduction

Bone marrow derived human mesenchymal stem cells (hMSCs) show great therapeutic potential in treatment of cardiovascular diseases. The protective function of hMSCs can be explained by secretion of antiapoptotic, angiogenic [1,2] and matrix-mediating factors [3]. Due to their multipotency, hMSCs are able to differentiate into endothelial like cells leading to an improved cardiac function [4]. Recently, microRNA (miR)—triggered modifications [5,6] have been shown to enhance efficiency of hMSC based therapy by influencing cell survival, proliferation, differentiation and production of paracrine factors [7]. miRs are key regulators of gene expression on the post-transcriptional level, as they control approximately 30% of all mammalian protein coding genes [8,9]. To date, numerous synthetic miRs, which mimic precursor or mature miRs are commercially available, however efficient and safe delivery methods suitable for clinical applications has not been developed, yet. Initially, research was concentrated on viral vectors, e.g., retroviruses and adenoviruses, as they provide high transduction efficiencies and long term gene expression. However, this approach has many disadvantages, such as toxicity, immunogenicity, mutagenicity, low genetic material load and high costs [10]. Therefore, a focus has been placed on alternative non-viral delivery approaches. Advantages of these methods include low toxicity, low immunogenicity, large capacity and easiness of production [11]. Cationic polymers represent a class of non-viral vectors with high transfection efficiency. One of the cationic polymers most successfully used both *in vivo* and *in vitro* is polyethylenimine (PEI) [12,13]. PEI contains a high density of amino groups, which provides its positive surface charge. Therefore, PEI is able to bind and protect negatively charged molecules, like miR, thereby forming complexes (polyplexes) through electrostatic interactions [14]. Furthermore, PEI polyplexes provide their efficient endosomal escape due to the so called “proton sponge effect”, avoiding degradation in lysosomes [15]. Previously, we have shown that a combination of PEI polyplexes with magnetic nanoparticles (MNP) via biotin-streptavidin connections (magnetic polyplexes) enables efficient transfection *in vitro. In vivo*, magnetic nanoparticles, carrying genetic material, can be targeted to the site of interest by external magnetic fields [16]. Advantages of this approach are decreased side effects, increased selectivity as well as reduced costs and dosage of the non-viral vector [17,18]. Moreover, cells transfected with magnetic nanoparticles have the potential to be targeted to the region of interest *in vivo* after transplantation into the organism, thus promoting the effect of cell-based therapies, e.g., in cancer treatment and cardiovascular diseases [19,20]. Initially, magnetic targeting of both viral and non-viral vectors has been introduced by Plank and his co-workers in 2002. They presented a novel method, where different vectors (e.g., Lipofectamine, PEI-DNA, recombinant adenovirus) were combined with paramagnetic nanoparticles via salt-induced aggregation. A static magnetic field was applied leading to increased sedimentation of the vectors on the cell surface with subsequent enhancement of transfection efficiency [21]. Since then, magnetically assisted transfection has been successfully used for efficient and rapid delivery of both, DNA [21,22] and siRNA [23,24] in different cell lines and human primary cells [18,25]. In order to further improve transfection rates McBain and Dobson have introduced a horizontally oscillating magnet array, which promoted more efficient endocytosis of magnetic vectors due to additional mechanical stimulation of the cell membrane [24,26,27]. Furthermore, we have recently shown for plasmid based complexes, that transfection efficiency was enhanced by conjugation of PEI complexes to MNPs even without application of magnetic field, as magnetic polyplexes provided a faster release of DNA into the cytosol compared to PEI polyplexes. Moreover, DNA/PEI/MNP transfection complexes did not pass the nuclear membrane due to strong biotin-streptavidin connections between PEI and MNPs while polyplexes were able to enter the nucleus [28]. Therefore, the transport and release mechanism of MNP bound polyplexes may be beneficial for miR delivery as opposed to DNA because miRs exert their function in the cytosol close to the nucleus.

**Figure 1:**
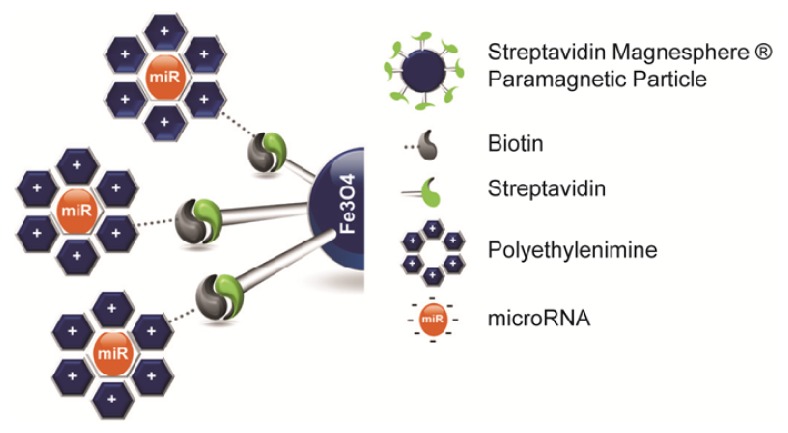
Schematic representation of magnetic transfection complexes Magnetic transfection complexes consist of streptavidin coated paramagnetic iron oxide nanoparticles in the core and miR/PEI polyplexes bound on them via streptavidin-biotin connections.

In this study, we developed a highly efficient non-viral vector for delivery of miR into hMSCs using miR/PEI/MNP complexes (Figure 1). Due to its critical role in hMSCs, we used miR-335, which is encoded in the second intron of the mesoderm-specific transcript (MEST) gene. It has been shown that miR-335 is regulating genes responsible for proliferation, differentiation and migration in hMSCs [29]. Moreover, miR-335 was found to be upregulated during myogenic differentiation *in vitro* and was induced during the regenerative phase after ischemia [30]. In this work we investigated the intracellular processing of precursor miR-335 to a mature strand as well as efficient knockdown of known target genes comparing the performance of PEI - mediated transfection and MNP-mediated transfection. Our results demonstrate that magnetic polyplexes provide a better long term effect, which is an important prerequisite for efficient genetic modifications of stem cells.

## 2. Results and Discussion

### 2.1. Optimization of Transfection Complexes in hMSCs

**Figure 2:**
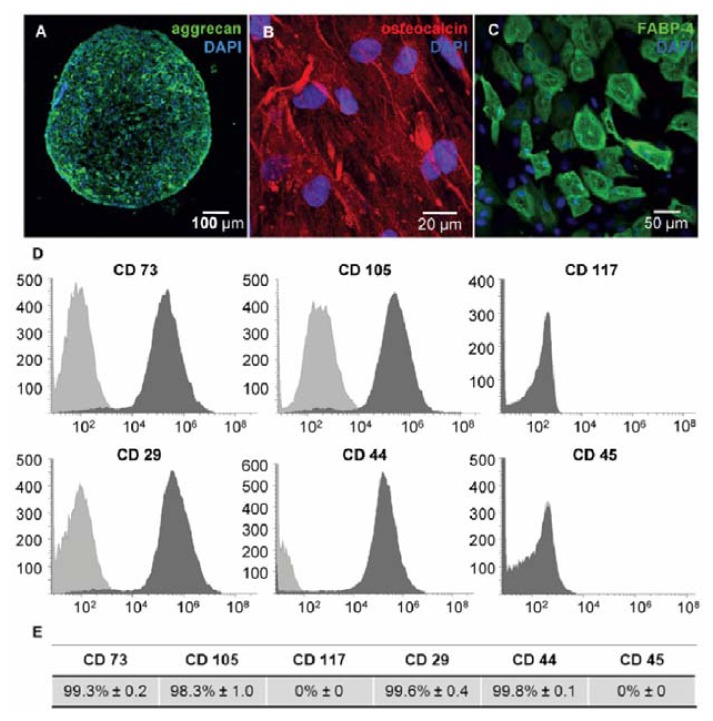
Characterization of hMSC (**A**–**C**) Differentiation capacity of hMSCs was shown by immunostaining of aggrecan (green) for chondrocytes (**A**), osteocalcin (red) for osteocytes (**B**) and FABP-4 (green) for adipocytes (**C**); Nuclei were stained with DAPI (blue); (**D**,**E**) Immunophenotyping of hMSCs was performed by flow cytometry after staining for specific CD surface markers. The bright gray areas indicate CD marker isotope controls (**D**); Surface marker expression values are in percentage of positive cells and represented as mean ± standard error, *n* = 5 (**E**).

hMSCs are defined as spindle shaped cells and can be characterized by specific surface marker expression and multilineage differentiation [31,32]. In respect to this definition, we isolated and characterized bone marrow derived hMSCs before use in further transfection experiments. The functionality of hMSCs was confirmed by differentiation of isolated cells towards chondrocytes (Figure 2A), osteocytes (Figure 2B) and adipocytes (Figure 2C). Moreover, the phenotype of hMSCs was verified by flow cytometry. As expected, isolated cells were positive for stem cell markers CD29, CD44, CD73 and CD105 but negative for hematopoietic markers CD45 and CD117 (Figure 2D,E).

**Figure 3:**
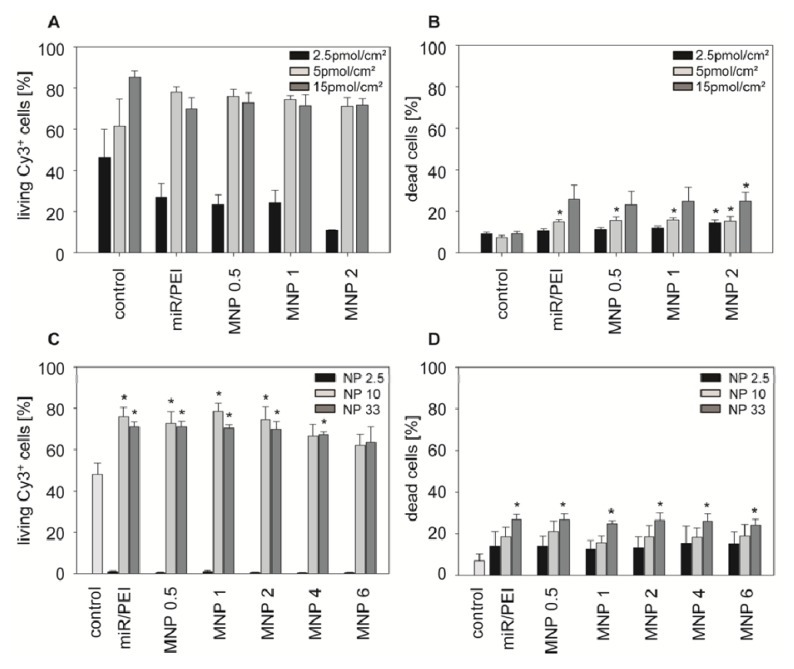
Transfection optimization with magnetic polyplexes in hMSCs. hMSCs were transfected with Cy™3 labeled miR/PEI or miR/PEI/MNP complexes and the uptake efficiency (**A**,**C**) and cytotoxicity (**B**,**D**) were determined by flow cytometry 5 h after transfection. (**A**,**B**) miR/PEI or miR/PEI/MNP complexes with various miR amounts (2.5, 5, 15 pmol/cm^2^ miR) at NP ratio 10 with MNP concentrations ranging from 0.5 to 2 μg/mL iron (MNP 0.5 to MNP 2); (**C**,**D**) Different NP ratios (NP 2.5, 10, 33) with varied MNP amount ranging from 0.5 to 6 μg/mL iron (MNP 0.5 to MNP 6). miR amount was kept constant (5 pmol/cm^2^ miR). Cells treated with naked miR were used as control. Values are presented as mean ± standard error, *n* = 3, *p ≤ 0.05 *versus* miR.

In order to optimize transfection efficiencies in hMSCs using magnetic miR/PEI/MNP complexes, we tested different miR amounts (2.5 to 15 pmol/cm^2^ miR) at NP ratio 10. Transfection complexes with 5 pmol/cm^2^ miR showed highly efficient uptake (~75%, Figure 3A) with relatively low cytotoxicity (~15%, Figure 3B). In contrast to previous publications, where higher miR amounts were used [33–35], an increase in miR amount did not lead to a further enhancement of complex uptake, but increased cytotoxicity (~25%, Figure 3B). Therefore, we decided to use 5 pmol/cm^2^ miR for the following experiments. To further improve uptake efficiencies, different NP ratios and MNP concentrations were tested and analyzed by flow cytometry while miR amount was kept constant (5 pmol/cm^2^ miR, Figure 3C,D). In our previous experiments with delivery of plasmid DNA using the same carrier system, we found that NP ratio 2.5 was optimal for transfection of hMSCs [28]. Nevertheless, miR and plasmid DNA differ in terms of structure, function and stability. It was shown by other groups, that NP ratios 10 and 33 were successfully used for delivery of siRNA using PEI complexes [33–37]. Therefore, as siRNA and miR have comparable size, structure and intracellular functionality, in our experiments we investigated NP ratios 2.5, 10 and 33. Nevertheless, in our experiments NP ratio 2.5 resulted in the lowest uptake rates (~1%, Figure 3C) whereas NP ratio 33 appeared to be toxic for cells (~25%, Figure 3D). In contrast, NP ratio 10 showed the highest uptake efficiency (Figure 3C), accompanied by good cell viability (Figure 3D). Therefore, we concluded that these conditions were optimal for efficient and safe miR delivery in hMSCs. We had observed a significant increase in uptake rates after transfection with miR/PEI/MNP complexes at low MNP dosage (0.5 to 2 μg/mL iron) compared to control (75% *vs.* 50%, Figure 3C). In addition, no significant differences in cytotoxicity compared to control were detected (Figure 3D). However, transfection complexes with higher MNP concentrations (4 to 6 μg/mL iron) did not lead to significant enhancement, indicating a limited uptake of these complexes or their insufficient stability. Therefore, we decided to use magnetic polyplexes containing 0.5–2 μg/mL iron for the following experiments. Although, miR/PEI/MNP and miR/PEI complexes performed in a similar manner, regarding their uptake efficiency and cell viability, magnetic complexes are beneficial for *in vivo* applications as they can be magnetically targeted to the tissues of interest in the organism [16]. Additionally, they can be monitored *in vivo* via magnetic resonance imaging (MRI) [38].

**Figure 4:**
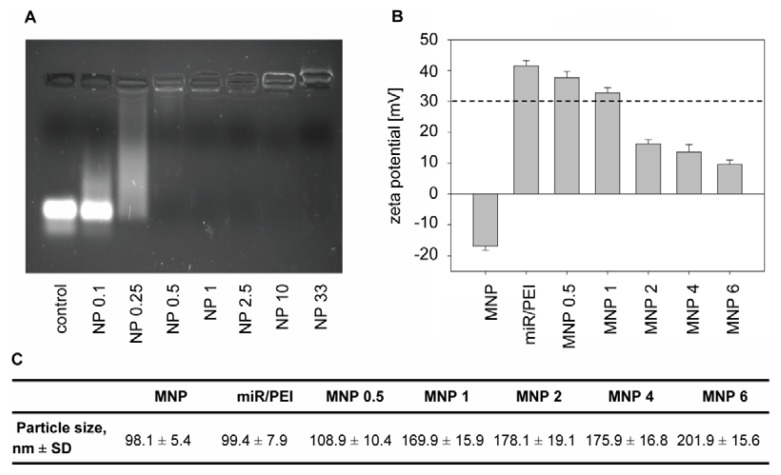
Characterization of transfection complexes. (**A**) Condensation of miR by PEI was examined by gel electrophoresis. Polyplexes with NP ratios from 0.1 to 33 and 20 pmol miR were investigated. miR alone was used as positive control. At NP ratio 0.5 the miR signal in the gel disappeared. Due to the bigger size of PEI complexes compared to miR alone, complexes do not run into the gel, but remain in the slots. This indicates a complete binding of miR to PEI; (**B**,**C**) Surface charge (**B**) and particle size (**C**) of MNP and transfection complexes were determined by DLS and PALS. Magnetic polyplexes with NP ratio 10, 20 pmol miR and MNP concentrations ranging from 0.5 to 6 μg/mL iron (MNP 0.5 to MNP 6) were used. Zeta potential data are presented as mean ± standard error, *n* = 10. Particle size data are presented as mean ± standard deviation, *n* = 10.

An important requirement for therapeutic applications of miR is the successful delivery to the cell and following release in the target compartment. To overcome the obstacles for efficient miR delivery our non-viral carrier was carefully designed with respect to physicochemical properties. At first, we studied condensation of miR by PEI using electrophoresis. As shown in Figure 4A, miR without PEI showed a strong and sharp main band under UV illumination. At NP ratio 0.25 this band had disappeared and the majority of the complexes migrated slower in the gel. At NP ratio 0.5 the miR signal in the gel disappeared entirely indicating that PEI complexes are not able to enter the gel, but remain in the slots. This can be explained by a complete binding of miR to PEI leading to complexes highly increased in size compared to miR alone. At our optimized NP ratio 10 (Figure 3C,D) PEI was able to condense all miR, thus protecting it from enzymatic degradation by nucleases [15,39]. Moreover, this tight condensation of miR can be beneficial due to masking of the double stranded RNA that is known to cause activation of the innate immune system [40].

Furthermore, particle size and surface charge of transfection complexes were investigated by Dynamic Light Scattering (DLS) and Phase Analysis Light Scattering (PALS), respectively, (Figure 4B,C) as these parameters have significant influence on the uptake mechanism [41,42]. In our experiments, sizes of transfection complexes ranged from 100 to 200 nm (Figure 4C). Previously, it has been shown that transfection complexes with particle sizes between 50 and 200 nm were optimal for an efficient endocytotic uptake into the cell [37,43]. Nevertheless, miR/PEI and miR/PEI/MNP complexes differed in surface charge. MNPs alone had a negative surface charge ranging from −18.35 to −15.61 mV. In contrast, miR/PEI complexes were strongly positively charged (41.54 ± 1.61 mV) indicating the presence of PEI on the surface, which is important for efficient endosomal escape (Figure 4B). It has been shown that PEI-containing complexes can escape the endosomes due to the “proton sponge effect”. PEI provides high buffering capacity and therefore is able to destabilize the endosomal membrane by osmotic swelling. The membrane of the endosome cannot resist and bursts releasing transfection complexes into the cytoplasm [44,45]. Moreover, high positive surface charge of complexes provides better binding to negatively charged cell membranes, facilitating subsequent cellular uptake [12]. Magnetic polyplexes with higher MNP concentrations (2 to 6 μg/mL iron) had a surface charge below +30 mV and therefore tended to build bigger complexes and aggregates [46,47]. However, miR/PEI/MNP complexes with 1 μg/mL iron showed sufficient positive surface charge (32.81 ± 1.76 mV), which provides optimal stability of transfection complexes in suspension. Regarding physicochemical properties, uptake efficiency and cytotoxicity of transfection complexes, 5 pmol/cm^2^ miR, NP ratio 10 and 1 μg/mL iron were considered to be the optimal parameters for magnetic complexes and thus used in all following experiments.

### 2.2. Monitoring of miR Processing in hMSCs over Time

**Figure 5:**
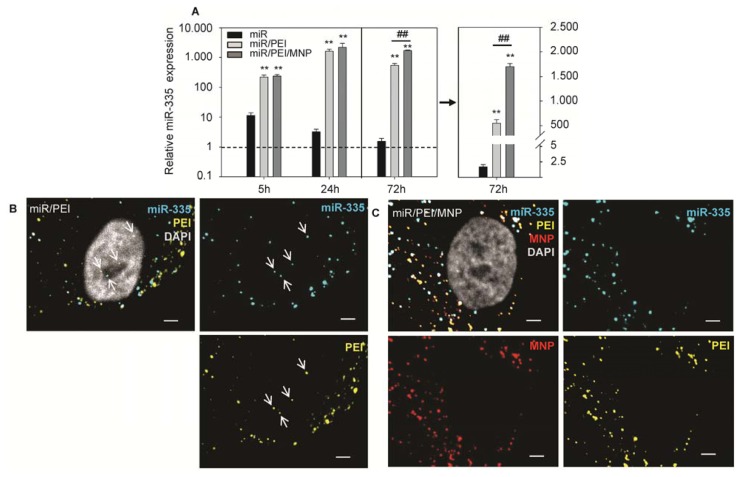
Processing of transfected precursor-miR. (**A**) hMSCs were transfected with precursor-miR-335 using miR/PEI or miR/PEI/MNP complexes and level of a mature miR-335 strand was detected by real time PCR 5, 24 and 72 h after transfection. Cells treated with miR only were used as a control. Dashed line indicates miR-335 expression in untransfected cells. Right plot shows a linear scale of miR-335 expression 72 h after transfection. Values were normalized to RNU6B expression and represented as mean ± standard error. The data are representative of 5 independent biological experiments (*n* = 5), each of which was measured in qPCR-triplicates. **p ≤ 0.001 versus miR, ^##^*p* ≤ 0.001 *versus* miR/PEI-mediated transfection; (**B**,**C**) Labeled miR/PEI (**B**) and miR/PEI/MNP complexes (**C**) were visualized by confocal laser scanning microscopy 72 h after transfection in hMSCs. miR-335 was labeled with Cy™5 dye (cyan), PEI was labeled with Oregon Green^®^ 488 (yellow) and MNPs were labeled with Atto 565 (red). Nuclei were counterstained with DAPI (gray). The arrow indicates condensed miR/PEI complexes inside the nucleus. Scale bar = 5 μm.

The mechanism of action and the functionality of the delivered miR are essential points to be considered for the development of an efficient miR carrier. In our study, we preferred delivery of precursor miR as it is more stable than mature miR and due to a high number of nucleotides it can be better condensed by PEI. The delivered precursor miR has to be processed inside the cell to a mature strand as previously described for endogenously expressed miR [9]. Furthermore, this processing step provides the basis for the subsequent RNA interference cascade [48]. Processing of precursor miR into mature strand was monitored 5, 24 and 72 h after transfection in hMSCs and relative expression of a mature miR-335 was quantified by real-time PCR (Figures 5A and S1; Table S1). A significant increase of miR-335 level was observed already 5 h after transfection. Maximal values of mature miR-335 were reached at 24 h time point. Cells transfected with miR/PEI and miR/PEI/MNP complexes showed more than 1000-fold enhancement in miR-335 level compared to cells transfected with miR alone. Interestingly, 72 h after MNP–mediated transfection miR-335 expression remained at the same level, whereas miR expression after PEI alone–mediated transfection was decreased and was more than 3-fold lower compared to magnetic polyplex transfection. This indicates a sustained effect of MNP–based transfection.

In order to explain, why MNP containing complexes provided better long term performance, we visualized magnetic polyplexes inside the cell 72 h after transfection and compared it to polyplex-mediated transfection. For intracellular visualization of transfection complexes, we fluorescently labeled all components of miR/PEI and miR/PEI/MNP complexes. Thereby, we took advantages of a labeling method for DNA containing transfection complexes, recently developed by our group. This technique does not affect transfection efficiency and cell viability [28]. Therefore, it is a reliable method for intracellular imaging of transfection processes. However, no differences in transfection performance between miR/PEI and miR/PEI/MNP complexes were observed on the intracellular level 5, 24 and 72 h after transfection (data not shown). However, Figure 5B demonstrates that condensed miR/PEI complexes were found inside the nucleus, which is in agreement with previous publications [16,37,49]. Yet, for effective gene knockdown cytoplasmic release of miR is required. Importantly, in case of MNP-mediated transfection, transfection complexes were distributed exclusively in the cytoplasm and the perinuclear region but not in the nucleus (Figure 5C), which may provide a better accessibility of miR to further processing.

**Figure 6:**
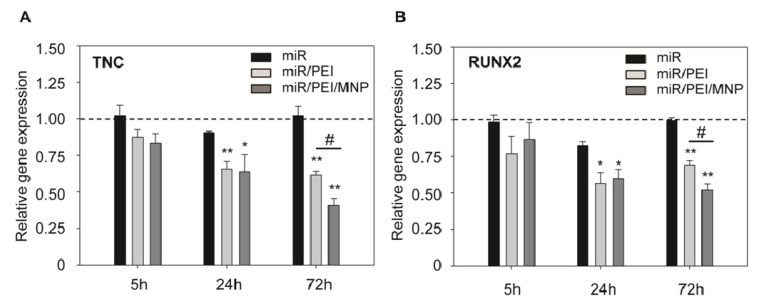
Efficient knockdown of miR-335 target genes. (**A**,**B**) hMSCs were transfected with miR/PEI or miR/PEI/MNP complexes and relative gene expression of TNC (**A**) and RUNX2 (**B**) was measured by real-time PCR 5, 24 and 72 h after transfection. Cells treated with miR only were used as control. Dashed line indicates gene expression in untransfected cells. Values were normalized to GAPDH expression and are represented as mean ± standard error, *n* = 5, *p ≤ 0.05 *versus* miR, **p ≤ 0.001 *versus* miR, ^##^*p* ≤ 0.001 *versus* miR/PEI - mediated transfection.

In order to test if the released and processed mature miR-335 is functional, we investigated the expression levels of its known target genes tenascin C (TNC) [50] and Runt-related transcription factor 2 (RUNX2) [29] 5, 24 and 72 h after transfection (Figure 6A,B; Tables S2 and S3). TNC is a protein of the extracellular matrix and was shown to be a strong modulator of cell proliferation and migration in cancer cells [51,52]. The transcription factor RUNX2 is a key regulator for osteogenic differentiation [29]. Transfection with unprotected miR-335, which was used as control, did not lead to sufficient knockdown of the investigated genes compared to untransfected cells even 72 h after transfection. That could be explained by low stability and fast degradation of naked miR by nucleases [53]. A remarkable knockdown of the investigated target genes was observed 24 h after transfection with both, miR/PEI and miR/PEI/MNP complexes, when compared to control. Interestingly, 72 h after transfection, TNC and RUNX2 mRNA levels were significantly downregulated after transfection with magnetic polyplexes as compared to PEI alone-mediated transfection, which confirms our hypothesis of a prolonged effect by MNPs.

**Figure 7:**
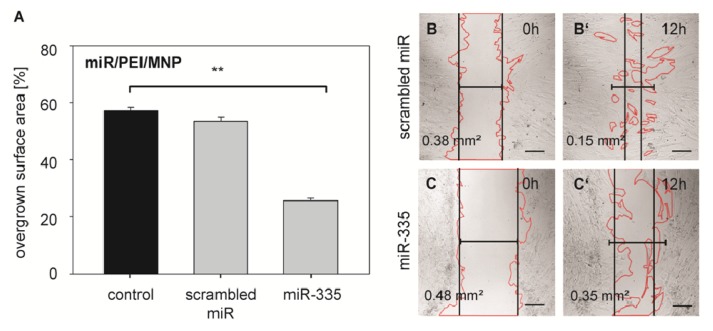
Migration activity of hMSCs after miR-335 transfection. (**A**) hMSCs were transfected with miR/PEI/MNP complexes and migration activity was tested 24 h after transfection. The overgrown surface area of transfected cells was measured before and 12 h after scratching. Untransfected cells were used as control. Data are presented as mean ± standard error, *n* = 5, **p ≤ 0.001; (**B**,**C**) Representative images after transfection with magnetic polyplexes containing either scrambled miR (**B**,**B′**) or miR-335 (**C**,**C′**). Images were taken immediately after (**B**,**C**) and 12 h after scratching (**B′**,**C′**). Values represent the non-overgrown surface area. Scale bar = 200 μm.

To confirm the efficient knockdown of TNC on the functional level of cell motility, migratory behavior of hMSCs after miR-335 transfection was investigated using an *in vitro* wound healing assay (Figure 7). Thereby, scrambled miR served as a control. miR/PEI/MNP complexes with scrambled miR (53.47% ± 1.55%) (Video 1) had no influence on cell motility compared to untransfected cells (57.16% ± 1.16%). However, after transfection with magnetic complexes using miR-335 (25.68% ± 0.87%) (Video 2) the migratory ability of hMSCs was reduced leading to a significantly lower surface area compared to untransfected control. The inhibition of cell migration by miR-335 is in good agreement with previous findings [50,54] and underlines that our magnetic non-viral vector is an ideal system to deliver and release miR, thereby efficiently blocking translation of target mRNAs. Therefore, our findings hold great promise for future *in vivo* applications in regenerative medicine.

## 3. Experimental Section

### 3.1. Culture of hMSCs

Bone marrow derived hMSCs were obtained from sternal aspirates of patients during coronary artery bypass grafting at the Cardiac Surgery Department of the University of Rostock as previously described [55]. The donors gave written consent to use their bone marrow for research purposes. Mononuclear cells were isolated by density gradient centrifugation. For plastic adherence selection cells were cultivated in Mesenchymal Stem Cell Growth Medium (MSCGM™, Lonza, Walkersville, MD, USA) containing 100 U/mL penicillin (PAA, Coelbe, Germany) and 100 μg/mL streptomycin (PAA) at 37 °C and 5% CO_2_. When the adherent hMSC population reached 80% confluency, cells were passaged or stored in liquid nitrogen. hMSCs in passage 3 and 4 were used in all experiments.

### 3.2. Immunophenotyping of hMSCs

Cell surface markers of hMSCs were fluorescently labeled with anti-human antibodies CD29-APC, CD44-PerCP-Cy5.5, CD45-V500, CD73-PE, CD117-PE-Cy7 (BD Biosciences, Heidelberg, Germany) and CD105-AlexaFluor488 (AbD Serotec, Kidlington, UK). Corresponding mouse isotype antibodies served as negative controls. 2 × 10^4^ cells were acquired using BD FACS LSRII™ flow cytometer (BD Biosciences, Heidelberg, Germany) and analyzed with BD FACSDiva Software 6 (BD Biosciences).

### 3.3. Functional Characterization for hMSCs

Differentiation capacity of hMSCs was investigated using Human Mesenchymal Stem Cell Function Identification Kit (R & D Systems, Minneapolis, MN, USA) according to the manufacturers’ protocol. After 21 days in differentiation medium, immunostaining of fatty acid binding protein-4 (FABP-4), osteocalcin and aggrecan for adipogenic, osteogenic and chondrogenic differentiation, respectively, was performed. Nuclei were stained with 4′,6-diamidino-2-phenylindol (DAPI, Invitrogen, Carlsbad, CA, USA). Samples were analyzed using ELYRA PS.1 LSM 780 microscope (Carl Zeiss, Jena, Germany) and ZEN2011 software (Carl Zeiss, Göttingen, Germany).

### 3.4. Preparation and Characterization of Transfection Complexes

Streptavidine Magnesphere^®^ Paramagnetic Particles (Promega, Madison, WI, USA) were sonicated and filtered using 450 nm Millix-HV PVDF syringe driven filter (Millipore, Tullagreen, Ireland). MNP filtrate was stored in aliquots at 4 °C. Cy™3 labeled Pre-miR™ Negative Control #1 (Ambion, Austin, TX, USA) was used for uptake efficiency studies; hsa-miR-335-5p Pre-miR™ miRNA Precursor (Ambion) and Negative Control #1 Pre-miR™ (Ambion) were used for functional studies. Branched polyethylenimine (MW = 25 kDa, Sigma-Aldrich, St. Louis, MO, USA) was biotinylated using Sulfo-NHS-LC-Biotin linker (Pierce, Rockford, IL, USA) according to the manufacturers’ protocol. Briefly, NHS-LC-Biotin linker was dissolved in DMSO at a final concentration of 9.7 mM. Afterwards, biotin was added dropwise to PEI (pH 6.4) and incubated for 16 h at room temperature in the dark. To remove the unreacted biotin, dialysis was performed and the concentration of α-amino groups in PEI was determined using 2% Ninhydrin reagent (Sigma-Aldrich). PEI was stored in aliquots at 4.41 mM amine concentration at 4 °C.

For optimization of complex composition different molar ratios of PEI nitrogen and miR phosphate (NP ratios) were prepared as previously described [2]. Briefly, miR and PEI were diluted in equal volumes of 5% glucose solution, mixed and incubated for 30 min at room temperature in order to form miR/PEI complexes. For miR/PEI/MNP complex formation, different iron concentrations of MNPs were mixed with miR/PEI complexes and incubated for 30 min at room temperature. Transfection complexes were freshly prepared before use.

The condensation of miR by PEI was investigated by gel electrophoresis. miR/PEI complexes were mixed with loading dye and loaded onto 2% agarose gel, containing ethidium bromide. An electric field of 100 V was applied for 15 minutes and image was taken using UV illuminator (Gel Doc 2000 system, Bio-Rad, Hercules, CA, USA).

The mean hydrodynamic diameter of MNPs and transfection complexes was measured using Dynamic Light Scattering (DLS) with Brookhaven 90 Plus Nanoparticle Size Analyzer (Brookhaven Instruments Corporation, New York, NY, USA). Zeta Potential was determined by Phase Analysis Light Scattering (PALS) using ZetaPALS Analyzer (Brookhaven Instruments Corporation, Holtsville, NY, USA).

### 3.5. Transfection

For transfection experiments, 1.5 × 10^4^ and 1 × 10^5^ cells per well were seeded in 24 and 6 well plate, respectively. 24 h after cell seeding, miR/PEI and miR/PEI/MNP complexes were freshly prepared as described above and added dropwise to the medium. 5 h after transfection cells were washed with PBS and fresh medium was supplied.

### 3.6. Uptake Efficiency and Cytotoxicity

For determination of uptake efficiency, hMSCs were seeded in 24 well plates and transfected as described above for 5 h. Afterwards, cells were washed with 1 M NaCl solution to remove transfection complexes attached to the cell membrane and detached with Trypsin-EDTA solution (PAA). To evaluate cytotoxicity, cells were stained with Near-IR LIVE/DEAD^®^ Fixable Dead Cell Stain Kit (Molecular Probes, Eugene, OR, USA) and fixed with 4% PFA (Sigma-Aldrich). 2 × 10^4^ cells were acquired using BD FACS LSRII™ flow cytometer (BD Biosciences) and analyzed with BD FACSDiva Software 6 (BD Biosciences, Heidelberg, Germany, 2007).

### 3.7. Fluorescent Labeling of Transfection Complexes

hsa-miR-335-5p Pre-miR™ miRNA Precursor (Ambion) was labeled with Cy™5 dye using Label IT^®^ miRNA Labeling Kit, Version 2 (Mirus Bio LLC, Madison, WI, USA) according to the manufacturer’s protocol. Briefly, 1 μg of miR was incubated with 8 μL of Label IT reagent for 2 h at 37 °C. Unreacted dye was removed using a purification column. Labeled miR-Cy5 was stored at −20 °C in the dark.

PEI was labeled using FluoReporter^®^ Oregon Green^®^ 488 Protein Labeling Kit (Molecular Probes). According to the manufacturers’ protocol, PEI was mixed with 1 M sodium bicarbonate solution. Afterwards Oregon Green^®^ stock solution (10 mg/mL in DMSO) was added to PEI solution and incubated for 1 h in the dark. The unbound dye was removed using a spin column. Labeled PEI-488 was stored at 4 °C protected from light.

MNP were labeled with Atto 565 dye conjugated to biotin (ATTO-TEC GmbH, Siegen, Germany) during miR/PEI/MNP complex formation. Therefore, miR/PEI complexes were mixed with MNPs and Atto 565 simultaneously and incubated for 30 min in the dark. Atto 565 dye was mixed with MNP at a ratio of 1:1000 (*w*/*w*). Labeled MNP-565 was freshly prepared before transfection.

### 3.8. Confocal Laser Scanning Microscopy

For microscopic observations, hMSCs were seeded on glass coverslips in 24 well plates and transfected with labeled complexes according to the optimized transfection protocol. miR-Cy5/PEI-488 and miR-Cy5/PEI-488/MNP-565 complexes with 5 pmol/cm^2^ miR, NP ratio 10 and 1 μg/mL of iron concentration within MNPs were used. 72 h after transfection cells were first washed with 1 M NaCl solution and then fixed with 4% PFA solution (Sigma-Aldrich) for 20 min at room temperature. Afterwards nuclei were stained with 250 nM DAPI (Molecular Probes) for 15 min at room temperature. Then, cells were washed with PBS and mounted with FluorSave™ Reagent (Calbiochem, Darmstadt, Germany) on microscope slides. Images were acquired in LSM mode using ELYRA PS.1 LSM 780 microscope and processed with ZEN 2011 Software (Carl Zeiss).

### 3.9. Real-Time PCR

For real-time PCR cells were seeded in 6 well plates and transfected with miR, miR/PEI or miR/PEI/MNP complexes as described above. 5, 24 and 72 h after transfection, total RNA was isolated with mirVana™ miRNA Isolation Kit (Ambion) according to the manufacturers’ protocol. Reverse transcription was performed using TaqMan^®^ MicroRNA Reverse Transcription Kit (Applied Biosystems™, Austin, TX, USA) and High Capacity cDNA Reverse Transcription Kit (Applied Biosystems™). Human mature miR-335 (Assay ID 000546), TNC (Assay ID Hs01115665_m1) and RUNX2 (Assay ID Hs00231692_m1) transcripts were quantified by StepOnePlus Real-Time PCR System (Applied Biosystems™) using the corresponding TaqMan Assays (Applied Biosystems™). To calculate the relative expression ratio (R) the ΔΔCt method was used (Equations (1) and (2)). Therefor RNU6B (Assay ID 001093) and Human GAPD Endogenous Control (Applied Biosystems™) were used as endogenous normalization controls for miR and protein coding genes, respectively. Untransfected cells were used as a reference. The obtained data are representative of 5 independent biological experiments (*n* = 5), each of which was measured in qPCR-triplicates.

(1) $$\Delta {\text{C}}_{\text{T}}={\text{C}}_{\text{T}}\;\text{target}-{\text{C}}_{\text{T}}\;\text{endogenous control}$$

(2) $$R=2^{-(\Delta {\text{C}}_{\text{T}\;\text{sample}}-\Delta {\text{C}}_{\text{T}\;\text{reference}})}$$

### 3.10. Wound Healing Assay

For functional studies, cells were seeded in 24 well plates and transfected with miR/PEI/MNP complexes according to the optimized transfection protocol as described above. Transfection complexes were formed either with hsa-miR-335-5p Pre-miR™ miRNA Precursor (Ambion) or with scrambled Negative Control #1 Pre-miR™ (Ambion). Twenty-four hous after transfection, fresh medium was added and a scratch was created in a cell monolayer with a sterile plastic tip. Live cell migration was recorded in a time-lapse video by sequential acquisition of images every 3 min using ELYRA PS.1 LSM 780 microscope (Carl Zeiss) for 12 h at 37 °C and 5% CO_2_. The overgrown surface area was measured with ZEN 2011 Software (version 8; Carl Zeiss: Göttingen, Germany, 2012) at the beginning and at the end of the assay.

### 3.11. Statistical Analysis

Statistical analyses in all experiments were performed using Student’s *t*-test. Particle size data are presented as mean ± standard deviation (SD). All other values are presented as mean ± standard error of the mean (SEM). A *p*-value < 0.05 was considered to be statistically significant.

## 4. Conclusions

In this study, we successfully developed a magnetic non-viral vector system for efficient miR delivery into hMSCs. Considering the entire transfection process comprising miR delivery, release, processing, knockdown of target genes and functional analysis, we concluded that MNP bound polyplexes show better long term performance than sole polyplexes, which is beneficial for effective genetic modifications of stem cells. Furthermore, we expect that our magnetic non-viral vector provides a tool for *in vivo* targeting of miR to specific regions inside the organism and therefore serve as a basis for innovative therapies in regenerative medicine.

## Supplementary Information
