# Supplementary material for: Innovative Strategy for MicroRNA Delivery in Human Mesenchymal Stem Cells via Magnetic Nanoparticles
Source: Int J Mol Sci. 2013 May 23;14(6):10710–26. doi: 10.3390/ijms140610710 (PMC3709698; doi:10.3390/ijms140610710)
Supplement: Supplementary file 1 [file ijms-14-10710-s001.zip › ijms-33043-supplementary/ijms-33043-Supplementary.pdf]

Supplementary Information

Figure S1. Cytotoxicity of transfection complexes over time. (**A**) hMSCs were transfected with miR only, miR/PEI or miR/PEI/MNP complexes and cytotoxicity was determined by flow cytometry 5, 24 and 72 h after transfection. miR/PEI or miR/PEI/MNP complexes with 5 pmol/cm^2 miR at NP ratio 10 with 1 µg/mL MNPs were used. Untransfected cells were used as control, n = 1; (**B,C**) Gating strategy for LIVE/DEAD® staining; (**B**) Positive control. hMSCs were incubated for 20 min with 4% PFA and subsequently stained with Near-IR LIVE/DEAD® Fixable Dead Cell Stain Kit. Red indicates dead cell population. Black indicates live cell population; (**C**) Representative image after transfection with magnetic miR/PEI/MNP complexes 24 h after transfection.

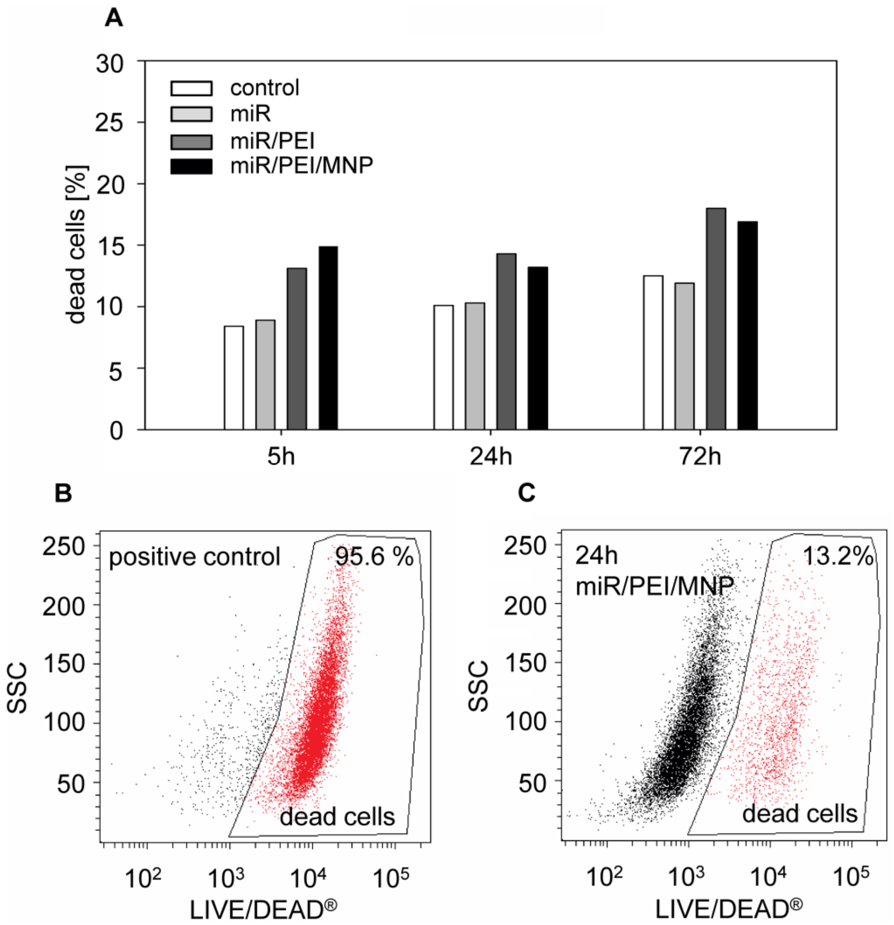

Table S1. Cycle threshold (Cт) values of mature miR-335 hMSCs were transfected with precursor-miR-335 using miR only, miR/PEI or miR/PEI/MNP complexes and level of a mature miR-335 strand was detected by real time PCR 5, 24 and 72 h after transfection. Values were normalized to RNU6B expression. Untransfected cells were used as a reference to calculate the relative expression ratio (*R*). The data are representative of 5 independent biological experiments (*n* = 5), each of which was measured in qPCR-triplicates.

Timepoint	Sample	No	Cт Mean		ΔCт	ΔΔCт	R
			miR-335	RNU6B	Mean	Mean	
5 h	miR	1	28,9852	33,5727	−4,5875	−3,2098	**9,2523**
		2	29,6175	34,1744	−4,5569	−2,9131	**7,5323**
		3	26,5860	32,6303	−6,0443	−4,0247	**16,2757**
		4	31,3356	35,8184	−4,4828	−3,3282	**10,0434**
		5	28,5902	34,1744	−5,5842	−3,2852	**9,7484**
	miR/PEI	1	23,8323	32,7630	−8,9307	−7,5530	**187,7923**
		2	22,6175	32,0630	−9,4455	−7,8017	**223,1276**
		3	21,8911	31,7946	−9,9035	−7,8838	**236,1854**
		4	24,1152	33,2235	−9,1084	−7,9538	**247,9256**
		5	22,1075	32,1632	−10,0557	−7,7567	**216,2721**
	miR/PEIMNP	1	23,7668	32,3371	−8,5703	−7,1926	**146,2780**
		2	22,3919	32,2204	−9,8285	−8,1847	**290,9662**
		3	21,7014	31,9340	−10,2326	−8,2129	**296,7171**
		4	21,7611	30,6993	−8,9382	−7,7836	**220,3406**
		5	22,1935	32,2204	−10,0269	−7,7279	**211,9950**
	untransfected	1	33,5814	34,9590	−1,3777		
		2	32,6198	34,2636	−1,6438		
		3	30,9557	32,9753	−2,0197		
		4	31,8105	32,9650	−1,1546		
		5	31,9646	34,2636	−2,2990		
24 h	miR	1	29,7904	35,9402	−6,1498	−1,6268	**3,0882**
		2	27,7001	32,7212	−5,0212	−2,3906	**5,2436**
		3	28,0608	32,3681	−4,3073	−1,2965	**2,4564**
		4	27,8005	32,3402	−4,5397	−1,4775	**2,7847**
		5	28,0007	32,7212	−4,7205	−1,4356	**2,7049**
	miR/PEI	1	16,6253	31,2990	−14,6737	−10,1506	**1136,6977**
		2	17,5141	30,9345	−13,4204	−10,7898	**1770,3299**
		3	18,1011	31,3014	−13,2003	−10,1896	**1167,7882**
		4	21,9596	36,3471	−14,3875	−11,3253	**2565,9884**
		5	16,7514	30,9345	−14,1831	−10,8982	**1908,4791**
	miR/PEIMNP	1	15,0983	30,2430	−15,1447	−10,6217	**1575,5732**
		2	18,0464	31,1964	−13,1500	−10,5194	**1467,7814**
		3	18,7202	31,8987	−13,1785	−10,1677	**1150,2566**
		4	21,9627	37,1464	−15,1837	−12,1215	**4455,8879**
		5	16,9085	31,1964	−14,2879	−11,0030	**2052,3159**
	untransfected	1	27,2145	31,7376	−4,5231		
		2	29,9215	32,5521	−2,6306		
		3	29,0101	32,0208	−3,0108		
		4	27,0642	30,1264	−3,0622		
		5	29,2672	32,5521	−3,2849		

Table S1. *Cont.*

Timepoint	Sample	No	C_T Mean		ΔCT Mean	ΔΔCT Mean	R
			miR-335	RNU6B			
**72 h**	miR	1	29,6888	32,1914	−2,5026	−0,0174	**1,0121**
		2	27,1232	30,9602	−3,8370	−1,4472	**2,7267**
		3	28,9918	31,4741	−2,4822	−0,7210	**1,6483**
		4	31,5005	34,7863	−3,2858	−0,7389	**1,6689**
		5	28,1681	31,2549	−3,0868	−0,2886	**1,2214**
	miR/PEI	1	20,6292	32,7292	−12,1000	−9,6148	**784,0311**
		2	21,4123	32,9245	−11,5122	−9,1224	**557,3231**
		3	21,7118	32,2124	−10,5006	−8,7393	**427,3613**
		4	18,7821	30,3560	−11,5739	−9,0270	**521,6752**
		5	20,9429	32,5443	−11,6014	−8,8031	**446,6960**
	miR/PEIMNP	1	20,2035	33,2752	−13,0717	−10,5865	**1537,6868**
		2	17,9000	30,9356	−13,0355	−10,6457	**1602,0423**
		3	18,6565	31,2571	−12,6006	−10,8394	**1832,1868**
		4	18,0018	31,2101	−13,2082	−10,6613	**1619,4590**
		5	18,8843	32,5848	−13,7005	−10,9022	**1913,8324**
	untransfected	1	30,1571	32,6423	−2,4852		
		2	30,1950	32,5848	−2,3898		
		3	29,9509	31,7121	−1,7613		
		4	29,8123	32,3593	−2,5469		
		5	28,4118	31,2101	−2,7982		

Table S2. Cycle threshold (Cт) values of TNC hMSCs were transfected with miR only, miR/PEI or miR/PEI/MNP complexes and relative gene expression of TNC was measured by real-time PCR 5, 24 and 72 h after transfection. Values were normalized to GAPDH gene expression. Untransfected cells were used as a reference to calculate the relative expression ratio (*R*). The data are representative of 5 independent biological experiments ($n = 5$), each of which was measured in qPCR-triplicates.

Timepoint	Sample	No	Cт Mean		ΔCт	ΔΔCт	R
			TNC	GAPDH	Mean	Mean	
5 h	miR	1	23,4147	20,9970	2,4178	−0,1952	**1,1449**
		2	22,2976	23,3384	−1,0407	−0,0232	**1,0162**
		3	21,1047	23,3411	−2,2364	−0,1202	**1,0869**
		4	24,3736	24,0232	0,3505	−0,2152	**1,1609**
		5	24,2939	23,1906	1,1033	0,0627	**0,9575**
	miR/PEI	1	23,4992	20,6985	2,8007	0,1877	**0,8780**
		2	23,3799	24,2263	−0,8464	0,1711	**0,8882**
		3	22,1691	23,8650	−1,6959	0,4203	**0,7473**
		4	24,5940	24,0233	0,5707	0,0050	**0,9966**
		5	25,0963	23,9497	1,1466	0,1059	**0,9292**
	miR/PEIMNP	1	27,7902	24,6983	3,0918	0,4788	**0,7176**
		2	22,1571	22,9641	−0,8070	0,2105	**0,8642**
		3	21,2687	22,9580	−1,6892	0,4269	**0,7438**
		4	23,5946	23,0465	0,5480	−0,0177	**1,0123**
		5	26,1930	24,9753	1,2177	0,1770	**0,8845**
	untransfected	1	24,4286	21,8156	2,6130		
		2	22,8603	23,8778	−1,0175		
		3	21,8675	23,9837	−2,1162		
		4	24,8210	24,2553	0,5657		
		5	23,6960	22,6553	1,0406		
24 h	miR	1	24,4887	23,5675	0,9211	0,1105	**0,9263**
		2	21,3965	19,6164	1,7801	0,1671	**0,8906**
		3	21,5093	19,6234	1,8859	0,1933	**0,8746**
		4	21,4170	19,5174	1,8996	0,1363	**0,9098**
		5	21,1952	19,7084	1,4868	0,1036	**0,9307**
	miR/PEI	1	25,2994	24,0382	1,2612	0,4506	**0,7318**
		2	24,7899	22,5643	2,2256	0,6126	**0,6540**
		3	24,0156	21,1066	2,9090	1,2164	**0,4304**
		4	23,8341	21,6489	2,1852	0,4220	**0,7464**
		5	22,7903	20,9375	1,8528	0,4696	**0,7221**
	miR/PEIMNP	1	24,8506	22,9895	1,8611	1,0504	**0,4828**
		2	27,7221	24,8993	2,8228	1,2098	**0,4323**
		3	26,8557	24,6042	2,2515	0,5589	**0,6788**
		4	26,7716	24,5556	2,2160	0,4528	**0,7306**
		5	26,8300	24,9351	1,8949	0,5117	**0,7014**
	untransfected	1	24,8496	24,0389	0,8107		
		2	20,4286	18,8156	1,6130		
		3	20,3793	18,6867	1,6926		
		4	20,4057	18,6424	1,7632		
		5	20,5008	19,1176	1,3832		

Table S2. *Cont.*

Timepoint	Sample	No	CT Mean		ΔCT Mean	ΔΔCT Mean	R
			TNC	GAPDH			
72 h	miR	1	24,0965	23,4980	0,5985	0,0857	**0,9424**
		2	24,9529	22,4269	2,5260	−0,0714	**1,0507**
		3	22,7903	22,4082	0,3821	−0,3114	**1,2409**
		4	22,7776	22,4305	0,3471	0,0550	**0,9626**
		5	20,9647	23,3573	−2,3926	−0,0580	**1,0410**
	miR/PEI	1	27,4484	26,3584	1,0900	0,5772	**0,6703**
		2	23,8260	20,6985	3,1275	0,5301	**0,6925**
		3	27,5431	25,9710	1,5721	0,8786	**0,5439**
		4	27,7921	26,7329	1,0592	0,7672	**0,5876**
		5	24,8112	26,3712	−1,5600	0,7746	**0,5845**
	miR/PEIMNP	1	24,2940	22,6432	1,6507	1,1379	**0,4544**
		2	28,5914	24,6983	3,8931	1,2958	**0,4073**
		3	26,7429	25,1812	1,5617	0,8682	**0,5478**
		4	26,3920	24,3844	2,0077	1,7156	**0,3045**
		5	24,4875	25,1323	−0,6448	1,6898	**0,3100**
	untransfected	1	23,2498	22,7370	0,5129		
		2	25,3343	22,7370	2,5974		
		3	23,4019	22,7085	0,6935		
		4	22,9903	22,6983	0,2920		
		5	20,4696	22,8042	−2,3346		

Table S3. Cycle threshold (CT) values of RUNX2 hMSCs were transfected with miR only, miR/PEI or miR/PEI/MNP complexes and relative gene expression of RUNX2 was measured by real-time PCR 5, 24 and 72 h after transfection. Values were normalized to GAPDH gene expression. Untransfected cells were used as a reference to calculate the relative expression ratio (*R*). The data are representative of 5 independent biological experiments (*n* = 5), each of which was measured in qPCR-triplicates.

Timepoint	Sample	No	CT Mean		ΔCT Mean	ΔΔCT Mean	R
			RUNX2	GAPDH			
5 h	miR	1	23,6412	20,9970	2,6442	0,0312	**0,9786**
		2	22,6052	23,3384	−0,7332	0,2844	**0,8211**
		3	21,1672	23,3411	−2,1739	−0,0577	**1,0408**
		4	24,5839	24,0232	0,5607	−0,0050	**1,0035**
		5	24,1584	23,1906	0,9678	−0,0729	**1,0518**
	miR/PEI	1	23,5074	20,6985	2,8089	0,1959	**0,8731**
		2	24,7829	24,2263	0,5566	1,5741	**0,3358**
		3	21,8043	23,8650	−2,0607	0,0555	**0,9623**
		4	24,5996	24,0233	0,5763	0,0106	**0,9927**
		5	25,5996	23,9497	1,6499	0,6092	**0,6555**
	miR/PEIMNP	1	27,1781	24,6983	2,4797	−0,1333	**1,0968**
		2	22,8806	22,9641	−0,0835	0,9340	**0,5234**
		3	20,8393	22,9580	−2,1187	−0,0025	**1,0018**
		4	23,5806	23,0465	0,5340	−0,0317	**1,0222**
		5	26,6099	24,9753	1,6346	0,5939	**0,6625**
	untransfected	1	24,4286	21,8156	2,6130		
		2	22,8603	23,8778	−1,0175		
		3	21,8675	23,9837	−2,1162		
		4	24,8210	24,2553	0,5657		
		5	23,6960	22,6553	1,0406		
24 h	miR	1	24,6099	23,5675	1,0423	0,2317	**0,8517**
		2	21,5563	19,6164	1,9399	0,3268	**0,7973**
		3	21,5209	19,6234	1,8975	0,2049	**0,8676**
		4	21,6143	19,5174	2,0969	0,3337	**0,7935**
		5	21,3955	19,7084	1,6871	0,3040	**0,8100**
	miR/PEI	1	25,6428	24,0382	1,6045	0,7939	**0,5768**
		2	26,0845	22,5643	3,5202	1,9071	**0,2666**
		3	23,3879	21,1066	2,2812	0,5886	**0,6650**
		4	23,9918	21,6489	2,3430	0,5797	**0,6691**
		5	22,8356	20,9375	1,8981	0,5149	**0,6998**
	miR/PEIMNP	1	24,5071	22,9895	1,5175	0,7069	**0,6126**
		2	27,8943	24,8993	2,9950	1,3820	**0,3837**
		3	26,9964	24,6042	2,3922	0,6996	**0,6158**
		4	27,0944	24,5556	2,5388	0,7756	**0,5842**
		5	26,6528	24,9351	1,7177	0,3345	**0,7930**
	untransfected	1	24,8496	24,0389	0,8107		
		2	20,4286	18,8156	1,6130		
		3	20,3793	18,6867	1,6926		
		4	20,4057	18,6424	1,7632		
		5	20,5008	19,1176	1,3832		

Table S3. *Cont.*

Timepoint	Sample	No	C_T Mean		ΔC_T Mean	$\Delta\Delta C_T$ Mean	R
			RUNX2	GAPDH			
72 h	miR	1	23,9932	23,4980	0,4953	−0,0176	**1,0123**
		2	25,0249	22,4269	2,5981	0,0007	**0,9995**
		3	23,1068	22,4082	0,6985	0,0051	**0,9965**
		4	22,7578	22,4305	0,3273	0,0353	**0,9759**
		5	20,9498	23,3573	−2,4075	−0,0729	**1,0519**
	miR/PEI	1	27,5602	26,3584	1,2018	0,6890	**0,6203**
		2	23,8707	20,6985	3,1722	0,5749	**0,6714**
		3	27,0167	25,9710	1,0456	0,3521	**0,7834**
		4	27,5187	26,7329	0,7858	0,4938	**0,7102**
		5	24,6379	26,3712	−1,7333	0,6013	**0,6592**
	miR/PEIMNP	1	23,8368	22,6432	1,1935	0,6807	**0,6239**
		2	28,6613	24,6983	3,9630	1,3656	**0,3881**
		3	26,9712	25,1812	1,7900	1,0965	**0,4676**
		4	25,4589	24,3844	1,0746	0,7825	**0,5813**
		5	23,7188	25,1323	−1,4134	0,9211	**0,5281**
	untransfected	1	23,2498	22,7370	0,5129		
		2	25,3343	22,7370	2,5974		
		3	23,4019	22,7085	0,6935		
		4	22,9903	22,6983	0,2920		
		5	20,4696	22,8042	−2,3346		

© 2013 by the authors; licensee MDPI, Basel, Switzerland. This article is an open access article distributed under the terms and conditions of the Creative Commons Attribution license (http://creativecommons.org/licenses/by/3.0/).
